# Supplementary material for: scTIGER: A Deep-Learning Method for Inferring Gene Regulatory Networks from Case versus Control scRNA-seq Datasets
Source: Int J Mol Sci. 2023 Aug 28;24(17):13339. doi: 10.3390/ijms241713339 (PMC10488287; doi:10.3390/ijms241713339)

International Journal of Molecular Sciences

MDPI

# SUPPLEMENTARY DATA

## This Supplementary File S1 includes Figure S1-S5 and Table S1-S3.

**Table S1.** The predicted GRNs of AR, ERG and PTEN in ECs under normal versus cancerous conditions.

| Source | Target | Regulation Type | PMID |
|---|---|---|---|
| AR | AHNAK2 | Upregulation | 25237629 |
| AR | ARF5 | Upregulation | |
| AR | BAG3 | Downregulation | 36370105 |
| AR | CAV2 | Downregulation | 36370105 |
| AR | CDC42EP4 | Upregulation | 27374120 |
| AR | CDC42SE1 | Downregulation | 33263276 |
| AR | CHP1 | Upregulation | 27374120 |
| AR | CNRIP1 | Downregulation | 27374120 |
| AR | DOCK4 | Upregulation | 27374120 |
| AR | EIF2S1 | Upregulation | 36370105 |
| AR | EPC1 | Downregulation | |
| AR | EZH1 | Upregulation | |
| AR | FBXO3 | Upregulation | [77] |
| AR | GABARAPL1 | Downregulation | 36370105 |
| AR | GCC2 | Downregulation | [78] |
| AR | GNAI3 | Downregulation | 36370105 |
| AR | GPM6A | Upregulation | 27374120 |
| AR | IDI1 | Downregulation | 25237629 |
| AR | KIF1B | Upregulation | 27374120 |
| AR | KRIT1 | Upregulation | 20634343 |
| AR | MMP24OS | Upregulation | |
| AR | MPRIP | Upregulation | 27374120 |
| AR | NOB1 | Upregulation | 33969736 |
| AR | NSUN6 | Downregulation | 36169095 |
| AR | NUMA1 | Downregulation | 10.1530/EO-22-0065 (DOI) |
| AR | PDCD7 | Upregulation | 27374120 |
| AR | PLA2G16 | Upregulation | |
| AR | PLS3 | Downregulation | 18511503 |
| AR | POGZ | Upregulation | 36370105 |
| AR | PPP2R1A | Upregulation | 36370105 |
| AR | PRNP | Downregulation | 25447311, 28915724 |
| AR | PSMA2 | Downregulation | |
| AR | REV1 | Upregulation | 36370105 |
| AR | ROBO1 | Upregulation | 27374120 |
| AR | S1PR1 | Downregulation | 32799825 |
| AR | TMEM184B | Downregulation | |
| AR | TMEM259 | Upregulation | 27374120 |
| AR | TMEM43 | Downregulation | 27374120 |
| AR | UBL3 | Downregulation | 27374120 |
| AR | XBP1 | Downregulation | 27374120 |
| AR | YTHDF3 | Upregulation | 34939643 |
| ERG | DDRGK1 | Downregulation | 16381927 |
| ERG | DPP8 | Downregulation | |
| ERG | HCLS1 | Downregulation | 27374120 |
| ERG | NDUFV2 | Downregulation | |
| PTEN | ACAP2 | Downregulation | 36370105 |
| PTEN | ALG5 | Upregulation | |
| PTEN | C1orf123 | Upregulation | |
| PTEN | GOLGA2 | Upregulation | 29921876 |
| PTEN | LAMA5 | Upregulation | 21731504 |
| PTEN | MGLL | Downregulation | 26997225 |
| PTEN | MYO6 | Downregulation | 36370105 |
| PTEN | NME4 | Upregulation | 36370105 |
| PTEN | RAB4A | Upregulation | 36370105 |
| PTEN | RNF10 | Upregulation | 36497344 |
| PTEN | S100A13 | Downregulation | |
| PTEN | S100A4 | Upregulation | 22740693 |
| PTEN | SAP30BP | Upregulation | |
| PTEN | SAT2 | Upregulation | |
| PTEN | SH3BGRL2 | Upregulation | [77] |
| PTEN | SHISA5 | Upregulation | |
| PTEN | SLC39A7 | Upregulation | 32109290 |
| PTEN | STX12 | Upregulation | 36370105 |

| Source | Target | Regulation Type | PMID |
|---|---|---|---|
| PTEN | TSR3 | Upregulation | |
| PTEN | TSTA3 | Upregulation | |
| PTEN | TTF1 | Upregulation | 28745797 |
| PTEN | TXNDC12 | Upregulation | |
| PTEN | ZC3H14 | Upregulation | |

**Table S2.** The predicted GRNs of AR, ATF3 and PTEN in prostate cancer between SMCs versus ECs.

| Source | Target | Regulation Type | PMID |
|---|---|---|---|
| AR | ADRM1 | Downregulation | 25237629 |
| AR | ATP2A2 | Downregulation | 19417088 |
| AR | CCT2 | Upregulation | 34680521 |
| AR | CDC42SE1 | Downregulation | 33263276 |
| AR | CHP1 | Upregulation | 27374120 |
| AR | CTBS | Upregulation | 36370105 |
| AR | CTGF | Downregulation | 27374120 |
| AR | EIF2S1 | Upregulation | 36370105 |
| AR | EPC1 | Downregulation | 27374120 |
| AR | EZH1 | Upregulation | |
| AR | FLOT1 | Downregulation | |
| AR | GATAD2B | Downregulation | |
| AR | GCC2 | Downregulation | [78] |
| AR | GDI1 | Upregulation | 18511503 |
| AR | GGCT | Upregulation | 27374120 |
| AR | GNAI3 | Downregulation | 36370105 |
| AR | HNRNPUL1 | Upregulation | |
| AR | ITFG1 | Upregulation | 27374120 |
| AR | KIF1B | Upregulation | 27374120 |
| AR | KRIT1 | Upregulation | 20634343 |
| AR | LATS2 | Upregulation | 36370105 |
| AR | MLH3 | Upregulation | 36370105 |
| AR | MMP24OS | Upregulation | |
| AR | MRPL36 | Upregulation | |
| AR | MRPS26 | Upregulation | 33348168 |
| AR | MYADM | Downregulation | |
| AR | NMT1 | Upregulation | 27374120 |
| AR | NOB1 | Upregulation | 33969736 |
| AR | NR2F2-AS1 | Upregulation | |
| AR | PDCD6 | Downregulation | 27374120 |
| AR | PET100 | Downregulation | |
| AR | POLE4 | Downregulation | 27374120 |
| AR | PPIP5K2 | Upregulation | 27374120 |
| AR | PPP4R2 | Upregulation | |
| AR | PSMA2 | Downregulation | |
| AR | PSMB4 | Downregulation | |
| AR | RPS17 | Upregulation | |
| AR | RWDD4 | Upregulation | |
| AR | SASH1 | Downregulation | 27374120 |
| AR | SDHD | Downregulation | 33709547 |
| AR | SETBP1 | Upregulation | 27374120 |
| AR | SMARCE1 | Upregulation | 36370105 |
| AR | SPART | Downregulation | |
| AR | SSBP2 | Upregulation | 27374120 |
| AR | STX8 | Upregulation | 27374120 |
| AR | TEAD1 | Downregulation | |
| AR | UBE2K | Downregulation | |
| AR | XBP1 | Downregulation | 36370105 |
| ATF3 | ACIN1 | Downregulation | 27374120 |
| ATF3 | C6orf89 | Downregulation | |
| ATF3 | CEP170 | Downregulation | 27374120 |
| ATF3 | CFI | Downregulation | |
| ATF3 | CLPTM1 | Upregulation | 27374120 |
| ATF3 | CNTLN | Downregulation | |
| ATF3 | DEGS1 | Downregulation | |
| ATF3 | ESF1 | Downregulation | 27374120 |
| ATF3 | FAM208A | Downregulation | |
| ATF3 | GLRX5 | Upregulation | 27374120 |
| ATF3 | HLTF | Upregulation | |

| Source | Target | Regulation Type | PMID |
|---|---|---|---|
| ATF3 | KIF1B | Downregulation | 27374120 |
| ATF3 | LTBP3 | Downregulation | |
| ATF3 | MKL2 | Downregulation | 27374120 |
| ATF3 | MRPL27 | Downregulation | 27374120 |
| ATF3 | PDCD7 | Downregulation | 27374120 |
| ATF3 | PSMC1 | Upregulation | 27374120 |
| ATF3 | RBM26 | Upregulation | |
| ATF3 | SMIM7 | Downregulation | 27374120 |
| ATF3 | VEZF1 | Downregulation | 27374120 |
| ATF3 | ZBTB8OS | Upregulation | 27374120 |
| ATF3 | ZC3H13 | Downregulation | 27374120 |
| ATF3 | ZNF791 | Upregulation | |
| HEXIM1 | ATF3 | Upregulation | 27374120 |
| RBM26 | ATF3 | Upregulation | |
| PTEN | ARHGEF1 | Downregulation | 36370105 |
| PTEN | CLEC2B | Downregulation | |
| PTEN | DPP8 | Downregulation | |
| PTEN | GLRX5 | Downregulation | 21371429 |
| PTEN | LAMB2 | Downregulation | 30478082 |
| PTEN | PRRC2A | Downregulation | |
| PTEN | SCAMP1 | Downregulation | 27374120 |
| PTEN | SNRPN | Downregulation | 22367183 |
| PTEN | TSPAN12 | Downregulation | |

**Table S3.** The predicted GRNs of BDNF, CREB1 and MAPK4 in neurons under fear conditioned versus non-fear control.

| Source | Target | Regulation Type | Literature Support |
|---|---|---|---|
| BDNF | IGFBP6 | Upregulation | 33673334 |
| BDNF | NPTX2 | Upregulation | 33383752 |
| BDNF | ACVR1C | Upregulation | 28927503 |
| BDNF | GRASP | Upregulation | 22396401 |
| IGFBP6 | RTN4RL2 | Upregulation | |
| CREB1 | ANO10 | Upregulation | 27374120 |
| CREB1 | FAM149A | Upregulation | 36370105 |
| CREB1 | DTX4 | Upregulation | 27374120 |
| CREB1 | GM45343 | Upregulation | |
| CREB1 | TUBB2B | Downregulation | 27374120 |
| CREB1 | GM7816 | Downregulation | |
| MAPK4 | PPT2 | Upregulation | |

**Figure S1. Combined GRNs of AR, PTEN and ERG.** For each gene (AR, PTEN, and ERG), the directly connected genes are displayed. The arrowheads and T-heads indicate the positive and negative regulation respectively.

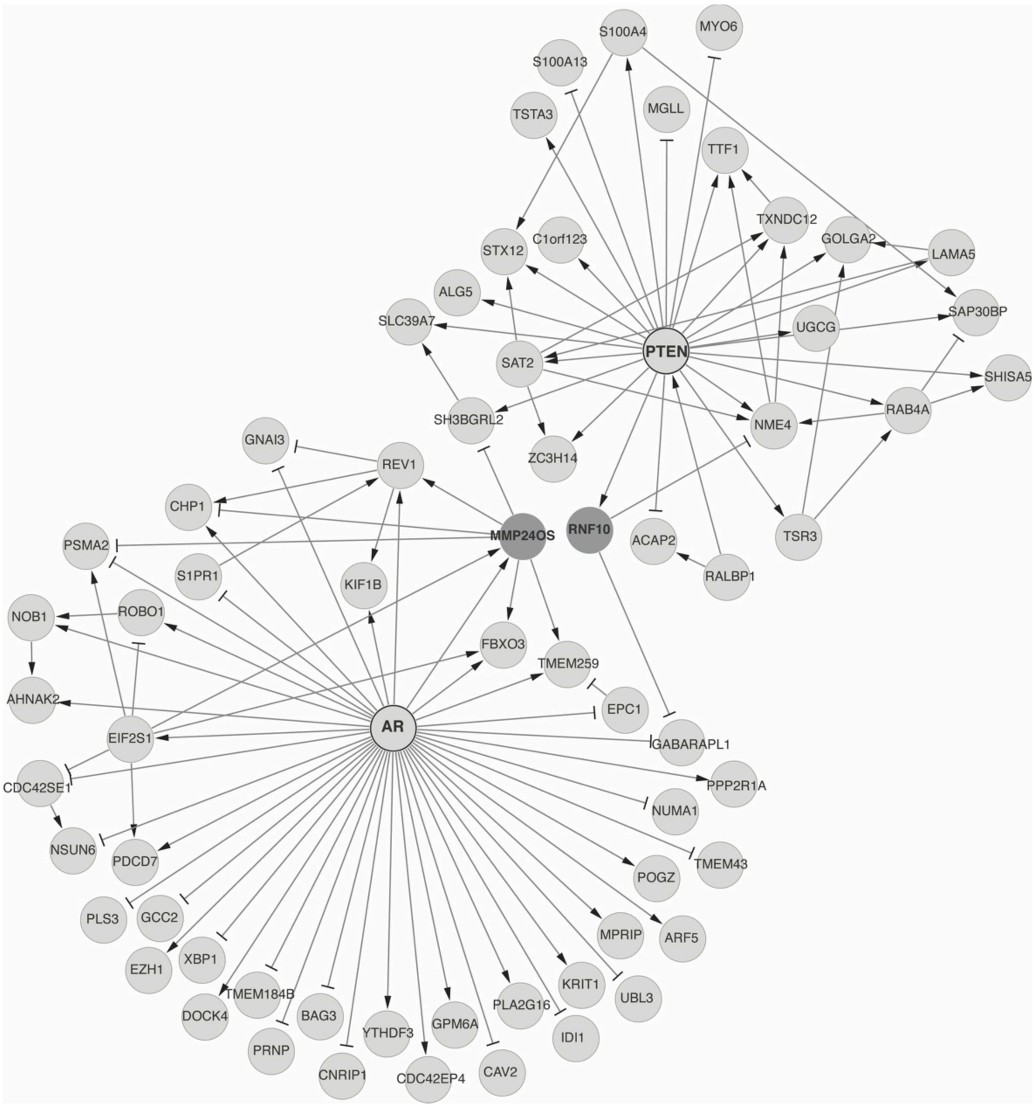

**Figure S2. Combined GRNs of AR, PTEN and ATF3.** For each gene (AR, PTEN, and ATF3), the directly connected genes are displayed. The arrowheads and T-heads indicate the positive and negative regulation respectively.

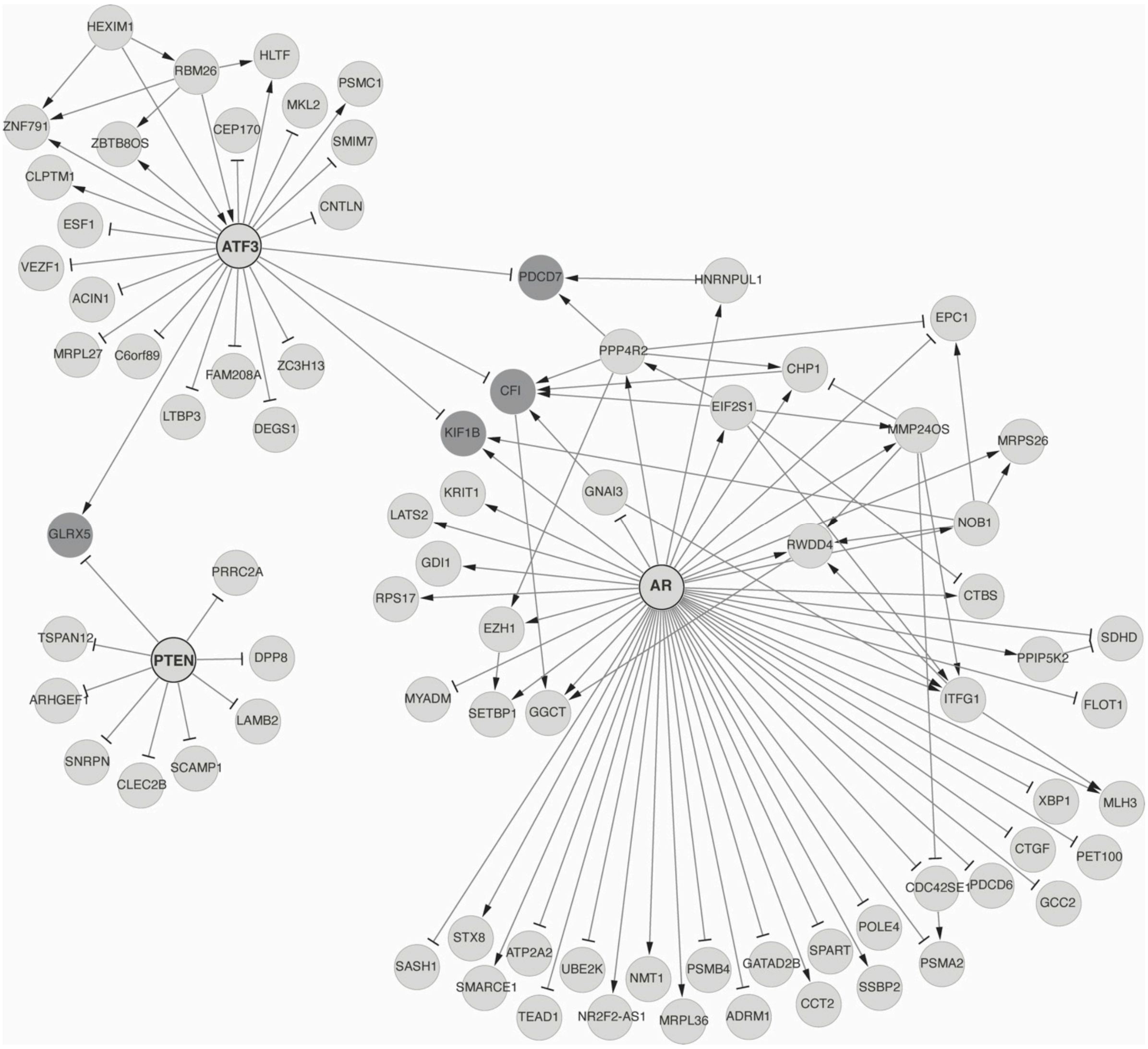

**Figure S3. Performance of scTIGER on different dropout rates.** The overlap rates were calculated for AR associated GRNs under two datasets of ECs and SMCs in prostate cancer.

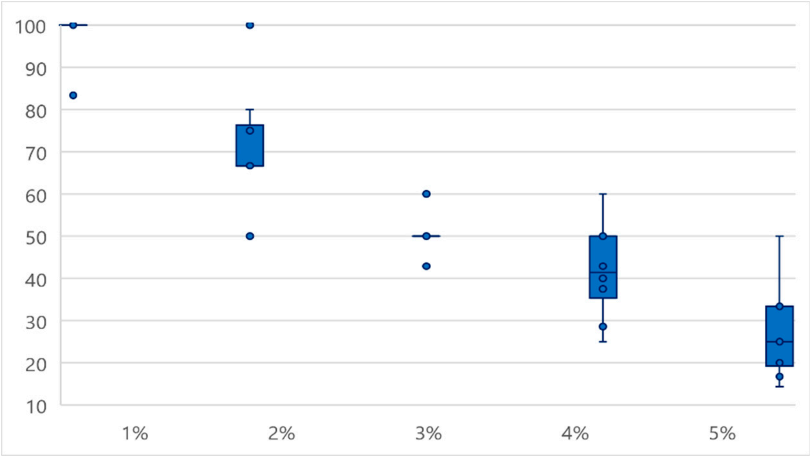


**Figure S4. Difference between detected interactions for ECs and SMCs by alternatively assigning them as case or control respectively**. Detected GRNs for **a**) ECs-SMCs and **b**) SMCs-ECs. Differing edges are shown with dashed lines and identical edges are shown with solid lines.

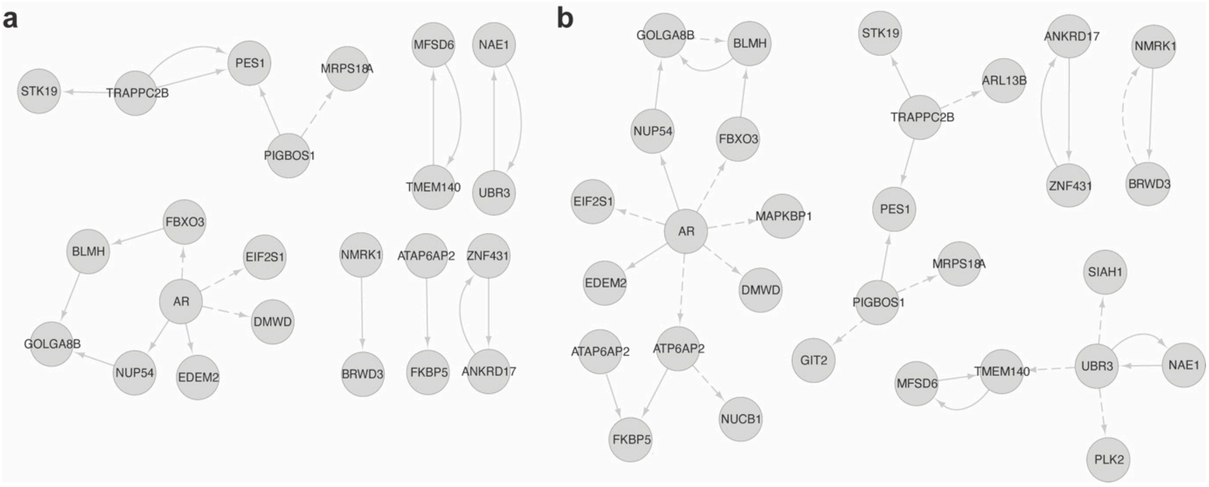

**Figure S5. The architecture examples of deep-learning approaches in scTIGER**. CNN: convolutional neuronal network.

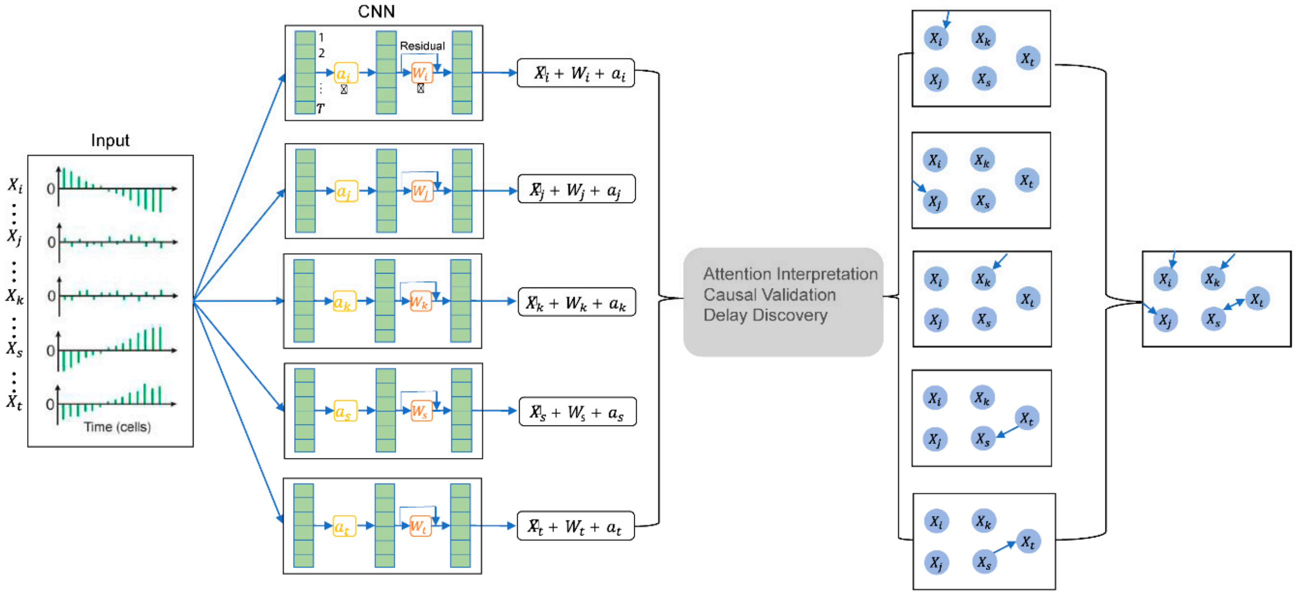

Supplement: Supplementary file 1 [file ijms-24-13339-s001.zip › ijms-2511507-supplementary.pdf]
